# Testis-Specific SEPT12 Expression Affects SUN Protein Localization and is Involved in Mammalian Spermiogenesis

**DOI:** 10.3390/ijms20051163

**Published:** 2019-03-07

**Authors:** Chung-Hsin Yeh, Ya-Yun Wang, Shi-Kae Wee, Mei-Feng Chen, Han-Sun Chiang, Pao-Lin Kuo, Ying-Hung Lin

**Affiliations:** 1 Division of Urology, Department of Surgery, Shin Kong Wu Ho-Su Memorial Hospital, Taipei 111, Taiwan; M000732@ms.skh.org.tw; 2 School of Medicine, Fu Jen Catholic University, New Taipei City 242, Taiwan; 053824@mail.fju.edu.tw; 3 Department of Chemistry, Fu Jen Catholic University, New Taipei City 242, Taiwan; vic0009@gmail.com; 4 Graduate Institute of Biomedical and Pharmaceutical Science, Fu Jen Catholic University, New Taipei City 242, Taiwan; shikae1017@gmail.com; 5 Bone and Joint Research Center, Chang Gung Memorial Hospital, Taoyuan 333, Taiwan; mfchen0@gmail.com; 6 Department of Obstetrics and Gynecology, College of Medicine, National Cheng Kung University, Tainan 701, Taiwan; 7 Department of Obstetrics and Gynecology, National Cheng Kung University Hospital, Tainan 704, Taiwan

**Keywords:** spermiogenesis, SEPT12, SPAG4

## Abstract

Male infertility is observed in approximately 50% of all couples with infertility. Intracytoplasmic sperm injection (ICSI), a conventional artificial reproductive technique for treating male infertility, may fail because of a severe low sperm count, immotile sperm, immature sperm, and sperm with structural defects and DNA damage. Our previous studies have revealed that mutations in the septin (SEPT)-coding gene *SEPT12* cause teratozoospermia and severe oligozoospermia. These spermatozoa exhibit morphological defects in the head and tail, premature chromosomal condensation, and nuclear damage. Sperm from *Sept12* knockout mice also cause the developmental arrest of preimplantation embryos generated through in vitro fertilization and ICSI. Furthermore, we found that SEPT12 interacts with SPAG4, a spermatid nuclear membrane protein that is also named SUN4. Loss of the *Spag4* allele in mice also disrupts the integration nuclear envelope and reveals sperm head defects. However, whether SEPT12 affects SPAG4 during mammalian spermiogenesis remains unclear. We thus conducted this study to explore this question. First, we found that SPAG4 and SEPT12 exhibited similar localizations in the postacrosomal region of elongating spermatids and at the neck of mature sperm through isolated murine male germ cells. Second, SEPT12 expression altered the nuclear membrane localization of SPAG4, as observed through confocal microscopy, in a human testicular cancer cell line. Third, SEPT12 expression also altered the localizations of nuclear membrane proteins: LAMINA/C in the cells. This effect was specifically due to the expression of SEPT12 and not that of SEPT1, SEPT6, SEPT7, or SEPT11. Based on these results, we suggest that SEPT12 is among the moderators of SPAG4/LAMIN complexes and is involved in the morphological formation of sperm during mammalian spermiogenesis.

## 1. Introduction

### 1.1. Mammalian Spermiogenesis and Male Infertility

Spermatogenesis and spermiogenesis are processes in which male germ cells undergo cell division through mitosis, meiosis, and differentiation. First, spermatogonia undergo mitosis and produce primary spermatocytes, which then undergo meiosis to generate round spermatids. Subsequently, the round spermatids undergo chromatin condensation, and shaping of the sperm head though forming the manchette, a transient microtubule structure. Finally, an elongated sperm tail is produced [1]. The regulatory pathways involved in the mentioned processes are largely unknown [2]. Infertility is a major health concern that affects nearly 1 in 10 couples, and half of the infertility cases can be attributed to male factors [3]. Disturbances in sperm shaping, defects in the sperm structure, premature chromosomal condensation, and DNA damage are considered to be the major causes of induced male infertility [4,5].

### 1.2. Roles of Septins in Mammalian Spermatogenesis

Septins (SEPTs) constitute the fourth component of the cytoskeleton; the other components are actin filaments, microtubules, and intermediate filaments. SEPTs are highly conserved GTP-binding proteins for polymerization [6]. The cellular roles of SEPTs include cytoskeletal remodeling, membrane compartmentalization, cell polarity establishment, cell cycle progression, and vesicle trafficking through interactions with several types of cytoskeletal proteins (e.g., tubulins, actin, and myosin II) [6]. SEPTs form complexes, which are used in various cellular functions [7,8]. In mammalian cells, 14 classes of *SEPTs* have been identified. Several *SEPT*-coding genes are specifically expressed in well-differentiated cells (e.g., neurons or male germ cells), whereas the others are universally expressed [9]. During spermatogenesis, SEPT4 plays a critical role in retaining the correct architecture of the mid piece and annulus, a ring-like structure between the mid piece and the principal piece of the flagellum, and in maintaining the appropriate structure of the mitochondria in the mid piece of the sperm tail [8,10]. Loss of the *Sept4* allele in mice causes immotile sperm with a defective annulus and midpiece, resulting in male sterility. In addition, we observed that SEPTs assemble as octameric filaments comprising SEPT proteins 12-7-6-4-4-6-7-12 and 12-7-6-2-2-6-7-12 in the annulus [11]. In *SEPT12*, it has been identified as a potential sterility-causing gene through cDNA microarray analysis, in which the testicular tissues of infertile and fertile men are compared [12,13]. During mammalian spermiogenesis, SEPT12 expresses around the manchette of elongating spermatids, the neck region of elongated spermatids, and the annulus of mature sperm. Loss of the *Sept12* allele in mice was revealed to result in distinctive morphological defects (e.g., abnormal sperm head, premature chromosomal condensation, nuclear damage, and bent tail) [13]. From a clinical aspect, *SEPT12* mutations in infertile men cause teratozoospermia and oligozoospermia [14,15].

### 1.3. Sperm Head Formation and SUN Proteins

During the shaping of the sperm head, the manchette, which is a transient microtubule-forming structure, provides strong force to remodel the nuclear envelope (NE), which is composed of an outer nuclear membrane and an inner nuclear membrane (INM) [16,17,18,19]. SUN and Nesprin proteins form the main structure of the linker of nucleoskeleton and cytoskeleton (LINC) complex, which connects the nucleoskeleton to the cytoskeleton. The SUN family includes SUN1–5, and most studies have focused on the functions of the nuclear membrane: SUN1 and SUN2 [20,21]. SUN1 or SUN2 interacts with LAMIN through the N-terminal domain and connects to the cytoplasmic actin through direct interaction with Nesprin. During the shaping of the sperm head, SUN3 is specifically expressed on the manchette in mice [19]. SPAG4 (SUN4) is localized at the manchette and axoneme and interacts with outer dense fiber protein 1 during the shaping of the sperm head and the elongation of the rat sperm tail [22]. In *Drosophila*, disruption of the *spag4* allele causes an abnormal morphology of the sperm head and dissociation of the centrioles from the nucleus in the sperm, resulting in male infertility [23]. Furthermore, *Spag4*^−/−^ mice have been reported to exhibit an irregular sperm head, damaged NE, and male infertility [24,25]. The phenotypes of *Spag4*^−/−^ mice on morphological formations of sperm are comparable with *Sept12^−/−^* mice. However, *Sept12* knockout mice exhibit severe phenotypes, such as decreased testis weight and sperm count. Loss of *Sept12* also increases the apoptotic signals of male germ cells [13].

### 1.4. SEPT12/SPAG4/LAMIN Complex

According to relevant studies, including our own, *Sept12* knockout and *Spag4* knockout produce similar results, namely impaired shaping of the sperm head and loss of tail elongation [13,24,25]. Specifically, the loss of *Spag4* in mice alters the distribution of the LINC proteins SUN1 and Nesprin 3, which play roles in spermatid elongation, and induces severe nuclear deformation [24,25]. Furthermore, SPAG4 was identified as a SEPT12 interactor through yeast-two hybrid screening [26]. However, whether SEPT12 affects SPAG4/LAMIN complexes in male germ cells remains unclear. In this study, we determined the effects of SEPT12 on the SPAG4/LAMIN complex. We found that SEPT12 alters the NE localizations of SPAG4/LAMIN complexes in male germ cells. The processes result from the expression of SEPT12 and not from that of SEPT1, SEPT6, SEPT7, or SEPT11. SEPT12 is important for SPAG4/LAMIN complexes during the differentiation of male germ cells.

## 2. Results

### 2.1. SEPT12 is Colocalized With SPAG4 During Murine Spermiogenesis

**Figure 1:**
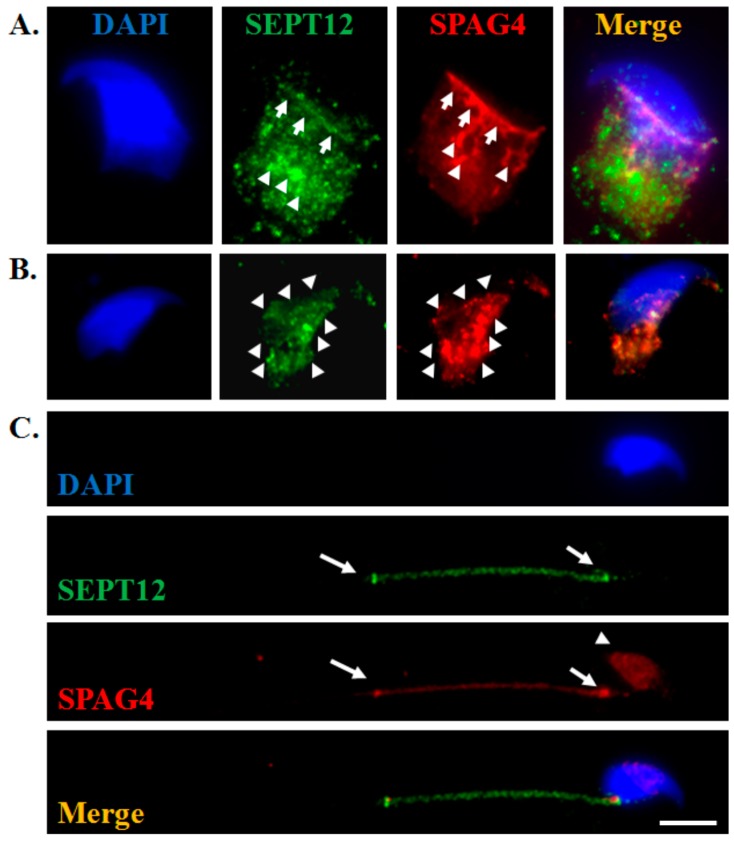
Colocalization of SEPT12 and SPAG4 during murine spermiogenesis. The testis collected from C57BL/6 mice (*n* = 3) were separated according to the male germ cells’ stages. The images at different developmental stages were captured, some of which are presented in the Figure. From left to right: DAPI staining (blue), SEPT12 signal (green), SPAG4 (red), and merging of DAPI, SEPT12, and SPAG4 at the elongating spermatids (**A**), elongated spermatids (**B**), and mature sperm (**C**). (**A**) Arrows indicate signals within the edges of the acrosome and manchette. Arrowheads indicate signals located at the manchette. (**B**) Arrowheads indicate signals within the manchette. (**C**) Arrows indicate signals located at the neck and annulus of mature sperm. Arrowheads indicate SPAG4 signals around the sperm head. Scale bar: 5μm.

To examine the dynamic localization between SEPT12 and SPAG4 at the different developmental stages of murine spermiogenesis, isolated murine male germ cells were subjected to an immunofluorescence assay. During the development of elongating spermatids, SEPT12 and SPAG4 started to colocalize at the rim between the edge of the acrosome and the manchette (Figure 1A, arrow) and were coscattered at the manchette (Figure 1A, arrowhead). Furthermore, SEPT12 and SPAG4 were partially colocalized at the manchette at the elongated spermatids (Figure 1B, arrowhead). In mature sperm, SEPT12 and SPAG4 were mainly colocalized at the centrosome and the annulus (Figure 1C, arrow). However, only SPAG4 was weakly localized at the acrosome (Figure 1C, arrowhead). According to these results, we suggest that SEPT12 may interact with SPAG4 during murine sperm head and tail formation.

### 2.2. SEPT12 Alters SPAG4 Localization

**Figure 2:**
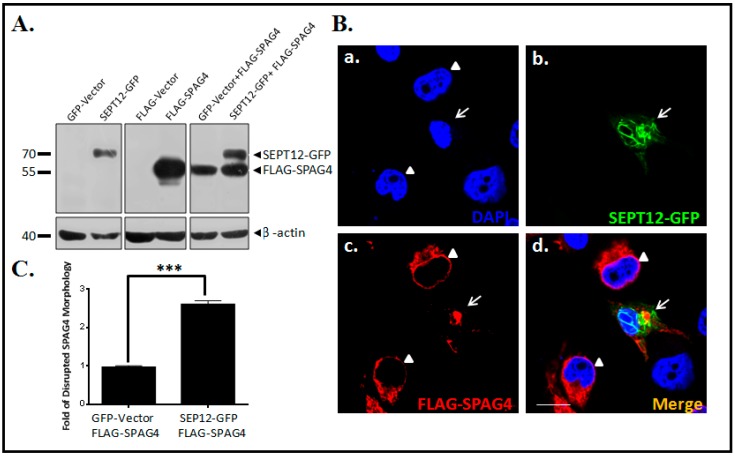
SEPT12 overexpression disturbs SPAG4 localization around the NE in the NT2/D1 male germ cell line. (**A**) Western blot analysis of NT2D1 cells transfected with the pEGFP-empty vector (Lane1; GFP-Vector), pEGFP-SEPT12 (Lane2; SEPT12-GFP), pFLAGP-empty vector (Lane3; FLAG-Vector), pFLAG-SPAG4 (Lane4; FLAG-SPAG4), a mixture of the pEGFP-empty vector and pFLAG-SPAG4 vector (Lane5; GFP-Vector + FLAG-SPAG4), and a mixture of the pEGFP-SEPTIN12 vector and pFLAG-SPAG4 vector (Lane6; SEPT12-GFP + FLAG-SPAG4) using the anti-EGFP and anti-FLAG antibodies. (**B**) Immunofluorescence staining of NT2/D1 cells cotransfected with the pEGFP-SEPTIN12 vector and pFLAG-SPAG4 vector using DAPI (blue) (a), anti-EGFP antibody (green) (b) and anti-FLAG antibody (red) (c); (d) image obtained after merging the images in (a), (b), and (c). Magnification ×400 in (a–d). The arrow indicates the cells transfected with the pEGFP-SEPTIN12 vector (green) and pFLAG-SPAG4 vector (red). The arrowhead indicates the cells that were transfected only with the pFLAG-SPAG4 vector (red). Scale bar: 10 µm. (**C**) Quantification of the disorganization of SPAG4 in the NT2D1 cells transfected with a mixture of the pEGFP-empty vector and pFLAG-SPAG4 vector (Bar 1; GFP-Vector + FLAG-SPAG4) and a mixture of the pEGFP-SEPTIN12 vector and pFLAG-SPAG4 vector (Bar 2; SEPT12-GFP + FLAG-SPAG4). At least 100 transfected cells were counted in each experiment. Two-tailed Student *t* test; error bars indicate ± standard error of mean (*** *p* < 0.0001).

To determine whether SEPT12 affects the NE localization of SPAG4 in human male germ cells, NT2/D1, a pluripotent human testicular embryonal carcinoma cell line was used. First, we constructed the pEGFP-SEPT12 and pFLAG-SPAG4 vectors and evaluated them through immunoblotting (Figure 2A). Second, the pEGFP-SEPT12 and pFLAG-SPAG4 vectors were cotransfected into NT2/D1 cells to determine the localization of SPAG4 in the cell line. When only SPAG4 was expressed in the cells, it was localized around the rim of the NE and the endoplasmic reticulum (ER; Figure 2B), which is consistent with the findings of previous studies [24,26]. However, expressed SEPT12 was determined to be formed in typical patterns and to particularly affect SPAG4 localization (Figure 2Bb,c,d), indicating that SEPT12 altered SPAG4 NE localization in the human male germ cell line.

### 2.3. Morphological Effects of SPAG4 on SEPT1/6/7/11

**Figure 3:**
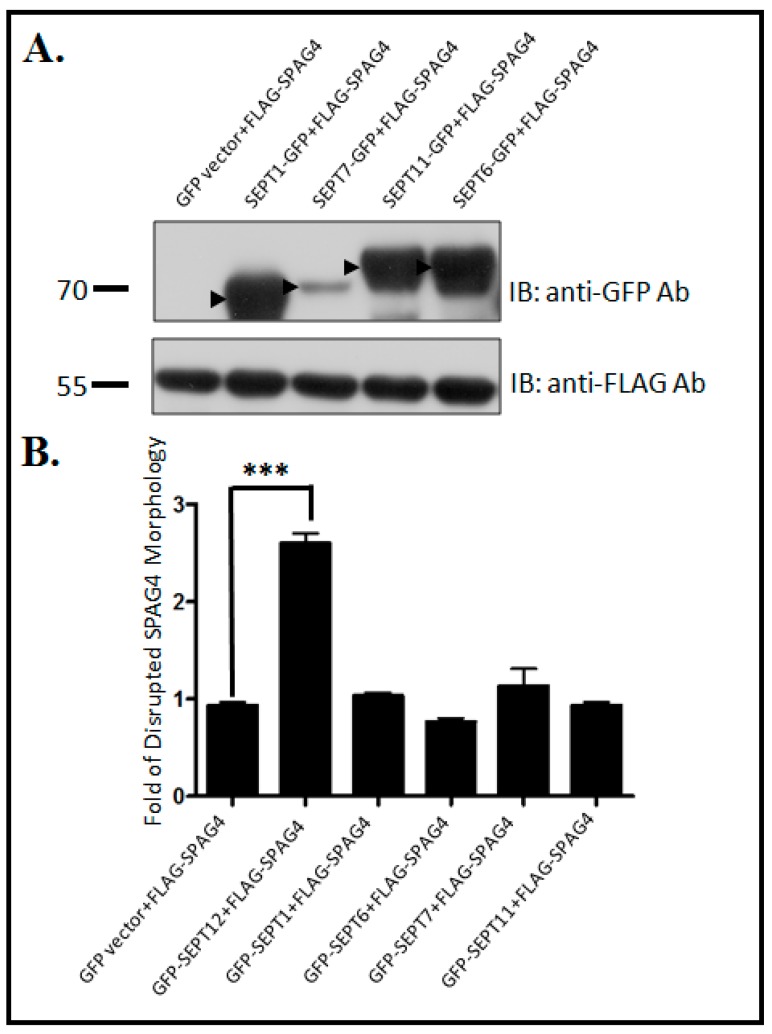
Effects of SEPT1/6/7/11 on SPAG4 localization around the NE in the NT2/D1 male germ cell line (**A**) Western blot analysis of NT2/D1 cells transfected with the pEGFP-vector, pEGFP-SEPT1, pEGFP-SEPT7, pEGFP-SEPT11, or pEGFP-SEPT6 with pFLAG-SPAG4 using the anti-GFP antibody (anti-GFP Ab) and anti-FLAG antibody (anti-FLAG Ab). (**B**) Fold changes in disrupted SPAG4 localization in transfected cells with the pEGFP-vector, pEGFP-SEPT12, pEGFP-SEPT1, pEGFP-SEPT6, pEGFP-SEPT7, or pEGFP-SEPT11 with pFLAG-SPAG4. The height of the boxes represents the mean of values obtained from three independent experiments. At least 100 transfected cells were counted in each experiment (*** *p* < 0.001, Student *t* test).

SEPT12 affected the NE localization of SPAG4, as indicated in Figure 2. However, to observe whether the phenomenon is specific for expressed SEPT12 or in general for SEPT overexpression, we selected SEPT1/6/7/11, which are universally expressed in cells, for further study. First, we constructed SEPT1/6/7/11 and evaluated their effects on SPAG4 localization. The SEPT1/6/7/11 proteins were expressed in NT2/D1 cells through immunoblotting (Figure 3A). Only the expression level of SEPT12 affected the distribution of SPAG4, whereas the expression levels of SEPT1/6/7/11 did not affect the SPAG4 distribution (Figure 3B). The aforementioned results suggest that only specifically expressed SEPT12 affected the NE localization of SPAG4 in the human male germ cell line.

### 2.4. Effects of SEPT12 on SPAG4/LAMIN complexes

**Figure 4:**
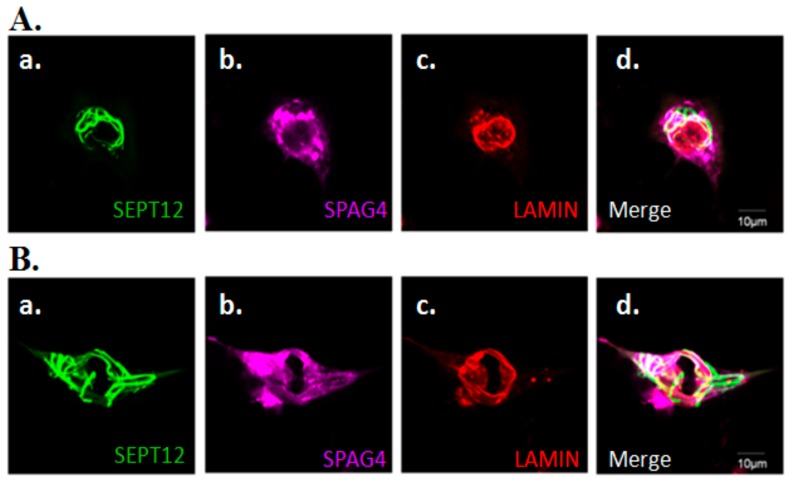
SEPT12 overexpression disturbs the morphology of the LAMIN in the NT2/D1 male germ cell line. SEPT12 signals are around NE (**A**) or cytoplasm (**B**). Immunofluorescence staining with the anti-LAMIN antibody in the NT2D1 cells cotransfected with the pEGFP-SEPTIN12 and pFLAG-SPAG4 vectors. (a) Anti-EGFP antibody (green), (b) anti-FLAG antibody (pink), (c) and anti-LAMIN antibody (red); (d) image obtained after merging the images in (a), (b), and (c). Scale bar: 10 µm.

**Figure 5:**
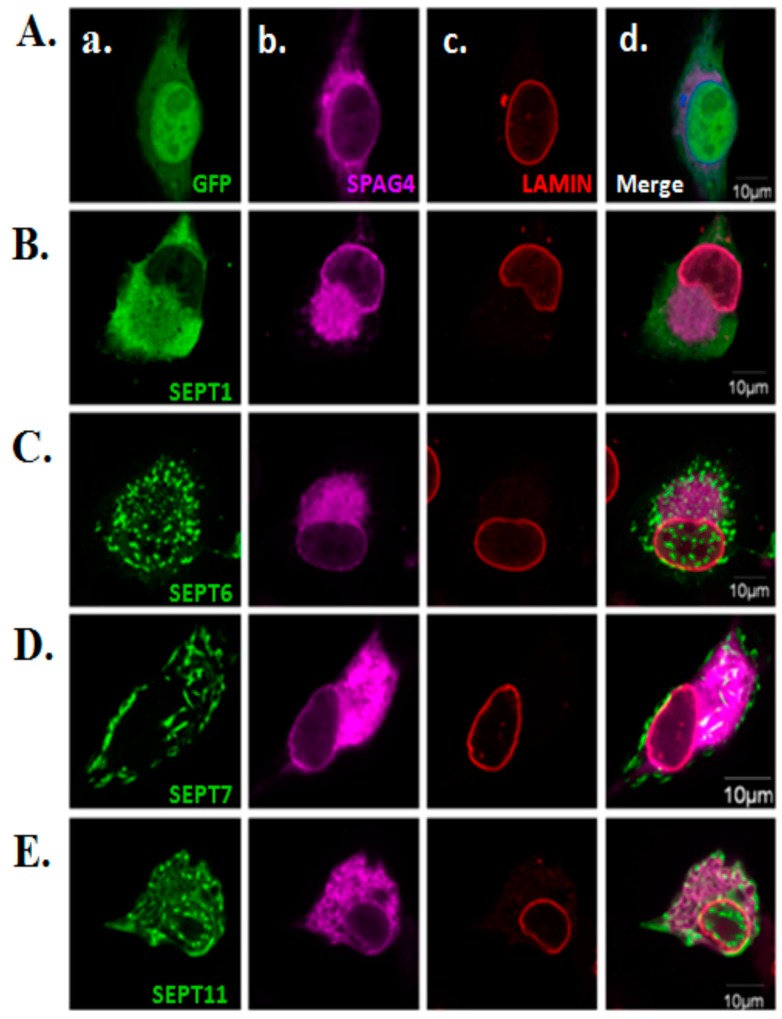
Effects of SEPT1/6/7/11 overexpression on the morphology of the NE in the NT2/D1 male germ cell line. Immunofluorescence staining with the anti-LAMIN antibody in the NT2D1 cells cotransfected with the pEGFP-empty (**A**), SEPT1 (**B**), SEPT6 (**C**), SEPT7 (**D**), or SEPT11 (**E**) vectors and pFLAG-SPAG4 vector. (a) Anti-EGFP antibody (green), (b) anti-FLAG antibody (pink), and (c) anti-LAMIN antibody (red); (d) image obtained after merging the images in (a), (b), and (c). Scale bar: 10 µm.

Within the INM, SUN proteins interact with LAMIN through its N-terminal domain [20]. Our previous study also revealed that SEPT12/SPAG4/LAMIN form complexes in NT2/D1 cells [26]. Based on these data, we suggest that SEPT12 is also required for the NE localization of the LAMIN complex. Hence, we determined that expressed SEPT12 influences the morphological structure of LAMIN when the cellular localization of SPAG4 is affected. Expressed SEPT12 formed typical patterns, one around NE (Figure 4Aa) and another around cytoplasm (Figure 4Ba). In these cells, expressed SEPT12 not only impaired SPAG4 but also affected the structure of LAMIN (Figure 4Ac,Bc). We also evaluated the effects of SPT1/6/7/11 on LAMIN and SPAG4 (Figure 5). Expressed SEPT1, SEPT6, SEPT7, and SEPT11 exhibited smear-, dot-, short-rod-, and dot-like patterns, respectively (Figure 5Aa,Ba,Ca,Da,Ea, respectively). SPAG4 and LAMIN were not affected by SEPT1/6/7/11 expression (Figure 5Ac,Bc,Cc,Dc,Ec). The aforementioned data indicate that SEPT12 altered the NE localization of SPAG4/LAMIN complexes.

## 3. Discussion

This is the first study to demonstrate that expressed SEPT12 alters the distribution of SPAG4, one of the SUN proteins, in human male germ cells. Furthermore, SEPT12 expression affects not only SPAG4 but also the NE localization of LAMIN. This phenomenon was determined to be due to only SEPT12 expression and not to SEPT1/6/7/11 expression. According to these results, we suggest that SEPT12 modulates the SPAG4/LAMIN complex and is involved in mammalian spermiogenesis.

### 3.1. Localization of SEPT12 is Similar to That of SPAG4 During Murine Spermiogenesis

Through electron microscopy, Shao et al. observed that rat SPAG4 was localized at the manchette and axoneme of elongating spermatids, and this is the first recognition of the expression pattern of SPAG4 [22]. Calvi et al. also identified that SPAG4 is closely associated with the manchette microtubules and suggested its role as a linker between the NE and manchette [24,25]. In this study, we observed that SEPT12 exhibited similar localization to SPAG4 at the manchette of murine spermatids. In addition, SEPT12 and SPAG4 were colocalized at the neck and annulus of murine mature sperm. However, the localizations and functions of SPAG4 and SEPT12 during human spermiogenesis remain limited. We propose that SEPT12/SPAG4 complexes are involved in murine sperm head and tail formations.

### 3.2. Localization of SPAG4 in Different Cell Models

In previous studies, SPAG4 have been transfected into HeLa cells and COS-7 cells and transiently localized to the NE and ER [24,25,27]. SPAG4 was localized around and along the nuclear rim. In addition, in these cells, SPAG4 interacted and colocalized with SUN3 around the nuclear rim and ER [24,25]. Because SEPT12 and SPAG4 are testis-specific proteins, we selected NT2/D1, a pluripotent human testicular embryonal carcinoma cell line, to evaluate the effects of SEPT12 on SPAG4/LAMINB complexes [26]. We found that overexpressed SEPT12 affected the localization of SPAG4 and the integration of LAMIN in NT2/D1 cells. This result suggests that the expression of SEPT12 modulates SPAG4/LAMINS complexes in human male germ cells.

### 3.3. Possible Roles of SEPT12/SPAG4/LAMIN Complexes During Sperm Head Shaping

Within the SUN family, SUN1 is primarily expressed during meiosis, and the loss of SUN1 in mice disrupts the link between the telomere and the NE, resulting in maturation arrest during meiosis [28]. Additionally, SUN4 colocalizes with LAMIN at the manchette of spermatids [24,26]. The loss of *Sun4* in mouse models also disturbs the integration of the NE of spermatids and results in the loss of sperm shaping. These results indicate the critical role of SPAG4 in the integration of the NE and in sperm head formation. In our series studies, we have found that the loss of *Sept12*/*SEPT12* function in mice and humans causes teratozoospermia and oligoospermia [13,15,29]. Furthermore, we found SEPT12 to show higher signals around the manchette and to interact with microtubules, which are the structural proteins of the manchette [30]. These reports indicate that SEPT12 is essential for sperm head shaping. We also found that SEPT12 interacts with SAPG4/LAMINB1 complexes [26]. In the present study, we found that SEPT12 expression affected the localization of SPAG4 and the integration of the NE in the human male germ cell line. Based on these results, we speculate that the SEPT12/SPAG4/LAMIN complexes are involved in sperm head shaping during mammalian spermiogenesis.

### 3.4. Teratozoospermia and LAMIN/SAPG4/SEPT12 Complexes

In the past 20 years, intracytoplasmic sperm injection (ICSI), a conventional artificial reproductive technique, has enhanced the success rate of paternity despite subfertility [31]. However, 25%–30% of infertile men cannot achieve parenthood through any of the usual therapies [32]. The major reason for sperm-related failure of ICSI is teratozoospermia (e.g., immature sperm and sperm with structural defects or with premature chromosomal condensation) [4,31]. In our previous studies, we have found that three mutated *SEPT12* sites, namely *SEPT12*^T89M^ (Thr89Met), *SEPT12*^D197N^ (Asp197Asn), and *SEPT12*^Del^ (c.474G/A-induced deleted form), caused teratozoospermia and oliogozoospermia [14,15]. SEPT12^D197N^ and SEPT12^Del^ exhibited a diminished ability to interact with SPAG4/LAMIN complexes compared with wild-type SEPT12 [26]. In the present study, we found that SEPT12 moderated the NE through altering SPAG4 and LAMIN in human male germ cells. We propose that LAMIN/SAPG4/SEPT12 complexes play a key role in shaping and maintaining the integration of the sperm head during spermiogenesis.

## 4. Experimental Section

### 4.1. Isolated Murine Testicular Germ Cells

The mouse study protocol was approved by the Institutional Animal Care and Use Committee of Fu Jen Catholic University (A10130; 13 August 2012). Testes were excised from 2-month-old C57BL/6 male mice (*n* = 3). Different developmental stages of male germ cells were isolated according to diverse centrifugal forces due to the different densities of the germ cells [14,15,16,17,18,19,20,21,22,23,24,25,26,27,28,29,30,31,32,33,34]. After decapsulation of the murine testes, the seminiferous tubules were cut into small pieces in DMEM/F12 medium, which comprised trypsin (1 mg/mL), collagenase (0.75 mg/mL), DNAase I (5 μg/mL), protease inhibitor cocktail (1X, Roche), and antibiotics (1×, Invitrogen). In addition, the enzyme digestion process was as follows: 1.5 h at 37 °C for 140 cycles per minute. These mixture solutions were filtered through 35-μM nylon filters to eliminate the undigested tissue. Four types of suspension cells mixtures were collected after centrifugation at 700, 400, 200, and 100× *g*. Finally, the suspension cells were collected after centrifugation at 3000× *g* and spread on a slide. Mature spermatozoa were collected from the vas deferens of the adult male mice.

### 4.2. Immunofluorescence Assay and Western Blotting

The immunofluorescence assay was performed according to our previous protocol [14,35,36]. The cells were treated with 0.1% Triton X-100, washed twice with Tris-buffered saline (TBS), and subsequently incubated with primary antibodies (SEPT12: Abnova, H00124404-B01P; SPAG4: Santa Cruz, sc-85927; GFP: Santa Cruz, sc-9996; FLAG: Sigma, F1804; and LAMINA/C: Santa Cruz, sc-20681) for 60 min at room temperature. After being washed with TBS, the sections were exposed to the Alexa Fluor 488 donkey antimouse IgG antibody (Invitrogen, cat no.A-21202, USA), Fluor 568 donkey antirabbit IgG antibody (Invitrogen, cat no. A-10042, USA), or Alexa Fluor^®^ 647 donkey anti-mouse IgG antibody (Invitrogen, cat no.A31571) for 60 min at room temperature and were washed again with TBS. 4′,6-Diamidino-2-phenylindole (DAPI; Invitrogen, USA, D3571) was used for staining the mitochondria and nuclei. The images at different developmental stages were captured, some of which are presented in the Figure 1. Western blotting was performed according to our previous protocol [36].

### 4.3. Cloning and Transfection

The full-length coding sequences of *SEPT1*, *SEPT6*, *SEPT7*, *SEPT11*, *SEPT12*, and SPAG4 were amplified from a human RNA panel by using reverse transcription polymerase chain reaction and were cloned into the pEGFP-N1 or pFLAG-CMV2 vector, as described previously [37]. All constructs were verified through DNA sequencing. Subsequently, the cell line was transfected with the vectors by using Lipofectamine (Invitrogen, USA), and the cells were analyzed using Western blotting or immunostaining.

### 4.4. Cell Culture

NTERA-2 cl.D1 (NT2/D1), a pluripotent human testicular embryonal carcinoma cell line, was used for Western blotting and immunostaining experiments. The base medium for the NT2/D1 culture consisted of Dulbecco’s modified Eagle’s medium (Catalog No. 11965-084; Gibco, NY, USA), 1X sodium pyruvate (Gibco), 10% fetal bovine serum (Sigma, MO, USA), and a 1X antibiotic–antimycotic solution (Caisson Laboratories, UT, USA). The cells were subcultured by scraping every 2–3 days.

## 5. Conclusions

In this study, we demonstrated that SEPT12 affects the localization of SAPG4 and LAMIN in human male germ cells, and this phenomenon is specifically due to SEP12 expression only and not to SEPT1/6/7/11 expression. Based on these results, we suggest that SEPT12 is a moderator of LAMIN/SPAG4 complexes during human sperm head and tail formation.
